# Muscimol Directly Activates the TREK-2 Channel Expressed in GABAergic Neurons through Its N-Terminus

**DOI:** 10.3390/ijms22179320

**Published:** 2021-08-27

**Authors:** Eun-Jin Kim, Oh-Sang Kwon, Chang-Gi Hur, Marie Merci Nyiramana, Dong-Kun Lee, Seong-Geun Hong, Jaehee Han, Dawon Kang

**Affiliations:** 1 Department of Physiology, College of Medicine and Institute of Health Sciences, Gyeongsang National University, Jinju 52727, Korea; eunjin1981@hanmail.net (E.-J.K.); realkosko@naver.com (O.-S.K.); mariemerci1994@naver.com (M.M.N.); dklee@gnu.ac.kr (D.-K.L.); hong149@gnu.ac.kr (S.-G.H.); jheehan@gnu.ac.kr (J.H.); 2 Cronex Co., Jeju 63078, Korea; lyden77@naver.com; 3 Department of Convergence Medical Science, Gyeongsang National University, Jinju 52727, Korea

**Keywords:** γ-aminobutyric acid, γ-aminobutyric acid receptor, two-pore domain K^+^ channel

## Abstract

The two-pore domain K^+^ (K_2P_) channel, which is involved in setting the resting membrane potential in neurons, is an essential target for receptor agonists. Activation of the γ-aminobutyric acid (GABA) receptors (GABA_A_R and GABA_B_R) reduces cellular excitability through Cl^-^ influx and K^+^ efflux in neurons. Relatively little is known about the link between GABA_A_R and the K^+^ channel. The present study was performed to identify the effect of GABAR agonists on K_2P_ channel expression and activity in the neuroblastic B35 cells that maintain glutamic acid decarboxylase (GAD) activity and express GABA. TASK and TREK/TRAAK mRNA were expressed in B35 cells with a high level of TREK-2 and TRAAK. In addition, TREK/TRAAK proteins were detected in the GABAergic neurons obtained from GABA transgenic mice. Furthermore, TREK-2 mRNA and protein expression levels were markedly upregulated in B35 cells by GABA_A_R and GABA_B_R agonists. In particular, muscimol, a GABA_A_R agonist, significantly increased TREK-2 expression and activity, but the effect was reduced in the presence of the GABA_A_R antagonist bicuculine or TREK-2 inhibitor norfluoxetine. In the whole-cell and single-channel patch configurations, muscimol increased TREK-2 activity, but the muscimol effect disappeared in the N-terminal deletion mutant. These results indicate that muscimol directly induces TREK-2 activation through the N-terminus and suggest that muscimol can reduce cellular excitability by activating the TREK-2 channel and by inducing Cl^-^ influx in GABAergic neurons.

## 1. Introduction

Neuronal activity is essential for developing and maintaining neuronal circuits and neurotransmitter switching in developing nervous systems [1]. Individual neurons display characteristic firing patterns determined by the numbers and types of ion channels and receptors in the membrane. Ion channels and receptors underlie electrical signals, and synaptic transmission switching occurs in the membrane over a long or short period of time [2]. The primary inhibitory neurotransmitter, γ-aminobutyric acid (GABA), activates the receptor, permitting Cl^−^ influx or K^+^ efflux. GABA receptor (GABAR) activation tends to maintain the resting membrane potential, making it more difficult for excitatory neurotransmitters to activate the neuron and create action potential.

Two-pore domain K^+^ (K_2P_) channels have the properties of background K^+^ channels and, therefore, play a crucial role in setting the resting membrane potential and regulating cell excitability. K_2P_ channels are modulated by various physical and chemical factors, such as membrane tension, lipids, volatile anesthetics, heat, oxygen, and protons [3]. Among members of the K_2P_ channel family, the TWIK-related acid-sensitive K^+^ (TASK; TASK-1 and TASK-3) and TWIK-related K^+^ (TREK)-1 and -2/TWIK-related arachidonic acid-stimulated K^+^ (TRAAK) (TREK/TRAAK) channels are modulated via stimulation of G-protein-coupled receptors (GPCR) [4]. Therefore, these channels represent important targets for receptor agonists that modulate cell excitability.

The TASK-1 and TREK-1 channels are expressed in GABAergic interneurons and regulated by GABA [5,6]. GABA acts primarily through the ionotropic receptor GABA_A_ and metabotropic receptor GABA_B_ to target cells. The activation of GABA_B_R, a GPCR, opens the K^+^ channels, and activation of the GABA_A_R opens the Cl^-^ channel, producing inhibitory signals in neurons. The TREK-2 channel is related to GABA_B_R-induced neuronal excitability and spatial learning inhibition in the entorhinal cortex [7]. However, little is known about links between the GABA_A_R and K^+^ channels. Moreover, the pharmacological, electrophysiological, and biochemical properties of GABA_A_R and GABA_B_R are different [8]. GABA_A_R, a part of the larger GABAR–Cl^-^ macromolecular channel complex, is the primary molecular target for general anesthetics [8]. Volatile anesthetics enhance GABA_A_R activity and activate the K_2P_ channels, leading to neuronal hyperpolarization and a reduction of excitability [9,10,11]. The relationship between the GABA_A_R and K_2P_ channels remains to be elucidated.

The present study was performed to identify expression of the K_2P_ channel in rat neuroblastic B35 cells and in GABAergic neurons obtained from transgenic mice expressing GAD67. B35 cells maintain glutamic acid decarboxylase (GAD) activity and express GABA [12] and GABA_A_R subunits [13], therefore it is possible to utilize them to study the effects of GABA_A_R modulators. The GABA_A_R agonist and antagonist effects on the K_2P_ channel’s expression and activity were analyzed in B35 cells and HEK-293 cells.

## 2. Results

### 2.1. Expression of the GABA Receptor and K_2P_ Channel mRNA in B35 Cells

**Figure 1:**
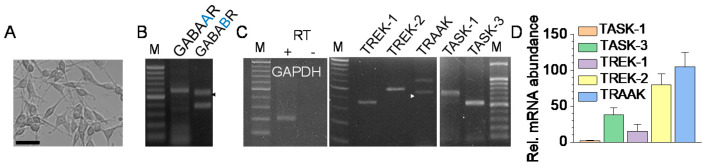
Expression of the K_2P_ channels in B35 cells. (**A**) A photomicrograph of B35 cells cultured in a 24-well culture dish. Scale bar, 50 μm. (**B**) Expression of GABA_A_ and GABA_B_ receptors. (**C**) Expression of TREK-1, TREK-2, TRAAK, TASK-1, and TASK-3 corresponding to the expected sizes of 361 bp, 493 bp, 445 bp, 702 bp, and 517-bp, respectively. The first, fourth, and tenth lanes presented 100 bp DNA ladders. RT + and RT – indicated, respectively, reactions with and without reverse transcriptase. The arrowheads indicate the GABA_B_ receptor and TRAAK bands. (**D**) Relative mRNA abundance of the K_2P_ channels. The mRNA expression levels of TASK-1, TASK-3, TREK-1, TREK-2, and TRAAK were quantified by real-time PCR. The mRNA expression level was normalized to GAPDH. Each bar represents the mean ± SD of four independent experiments.

B35 cells observed under a microscope (400×) showed neurite outgrowth (Figure 1A). B35 cells expressed the mRNAs of TASK (TASK-1 and TASK-3) and TREK/TRAAK (TREK-1, TREK-2, and TRAAK) channels (Figure 1C), as well as those of the GABA receptors (GABA_A_R and GABA_B_R) (Figure 1B). Real-time PCR data showed that the TREK-2 and TRAAK mRNA expression levels were higher than those of other channels (TASK-1, TASK-3, and TREK-1). The mRNA levels of TREK-2 and TRAAK were, respectively, 70 and 100 times higher than those of TASK-1 (Figure 1D). Subsequent experiments focused on the TREK/TRAAK channel because the TREK/TRAAK channels’ expression levels were relatively higher than the expression level of the TASK channel.

### 2.2. Localization of TREK/TRAAK Proteins in B35 Cells and GABAergic Neurons

**Figure 2:**
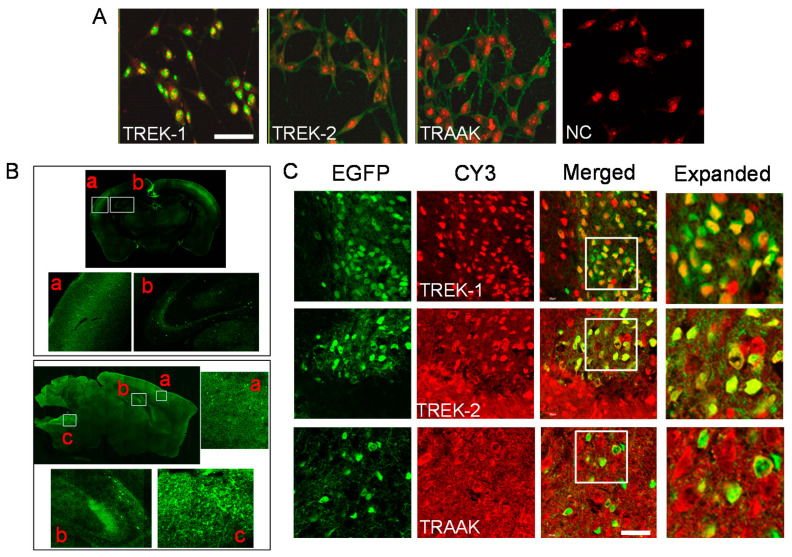
Expression of TREK/TRAAK in GABAergic neurons. (**A**) Localization of TREK-1, TREK-2, and TRAAK proteins in B35 cells. Cells were immunostained with anti-TREK-1, -TREK-2, or -TRAAK antibodies, and subjected to propidium iodide (PI) staining for nuclear staining. The merged image was output as an overlay of green fluorescence (FITC) and red PI-stain images. In the negative control (NC) group, the primary antibody was omitted. (**B**) The expression pattern of GABAergic neurons in the brain obtained from the GAD67-EGFP transgenic mice. The upper panel and lower panel present coronal section and sagittal section images, respectively. In the coronal and sagittal section images, **a**, **b**, and **c** represent the cortex, hippocampus, and medulla, respectively. (**C**) Co-localized TREK/TRAAK signals and GAD67-GFP signals in the medulla. GABAergic neurons expressed EGFP in green. The indicated antigen was immunostained red using CY3. EGFP and CY3 signals were merged in yellow (merge). An expanded view of the white box in the merged column is presented on the right side. Scale bar, 40 μm.

B35 cells expressed TREK-1, TREK-2, and TRAAK proteins. TREK-1 proteins were strongly localized to the nucleus, while TREK-2 and TRAAK proteins were predominantly expressed at the cell membrane (Figure 2A). GABAergic neurons were distributed in the cerebral cortex, hippocampus, and medulla of GAD67-EGFP mice (Figure 2B). As shown in Figure 2C, TREK and TRAAK were expressed in the GABAergic neurons in the medulla of GAD67-EGFP mice. TREK-1 was localized to the nucleus, while TREK-2 and TRAAK were evenly expressed throughout the cell (Figure 2C). These results are consistent with the expression patterns of TREK/TRAAK in B35 cells.

### 2.3. TREK-2 Expression Levels Increased by GABA Receptor Agonists in B35 Cells

**Figure 3:**
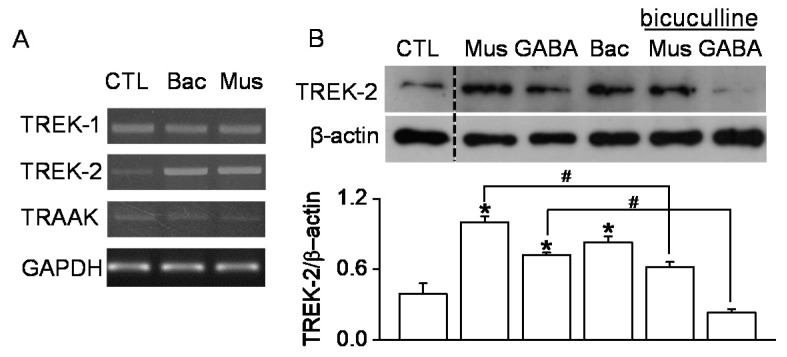
Upregulation of TREK-2 by the GABA receptor agonists baclofen and muscimol in B35 cells. (**A**) The alteration of TREK-1, TREK-2, and TRAAK mRNA expression levels via treatment with baclofen or muscimol. B35 cells were treated with the chemicals (100 μM) for 24 h. (**B**) The TREK-2 protein expression level affected by GABAR modulators. Each bar represents the mean ± SD of four independent experiments. * *p* < 0.05 compared to the control. ^#^ *p* < 0.05 compared to each corresponding treatment. CTL, Bac, and Mus represent the control, baclofen, and muscimol, respectively.

To investigate whether the TREK/TRAAK expression level is affected by GABAR agonists, the GABA_A_R agonist muscimol and GABA_B_R agonist baclofen were treated to B35 cells at a concentration of 100 μM for 24 h. Baclofen and muscimol markedly increased TREK-2 mRNA but not TREK-1 and TRAAK mRNA (Figure 3A). As shown in Figure 3B, the TREK-2 expression level was significantly increased compared to control by the GABAR agonists GABA, baclofen, and muscimol. Moreover, the TREK-2 expression level was significantly decreased in the presence of bicuculine, a GABA_A_ receptor antagonist, compared to each corresponding GABAR agonist (*n* = 4, *p* < 0.05). TREK-2 protein expression was more strongly upregulated by muscimol than by baclofen (Figure 3B).

### 2.4. Activation of TREK-2 by Muscimol

**Figure 4:**
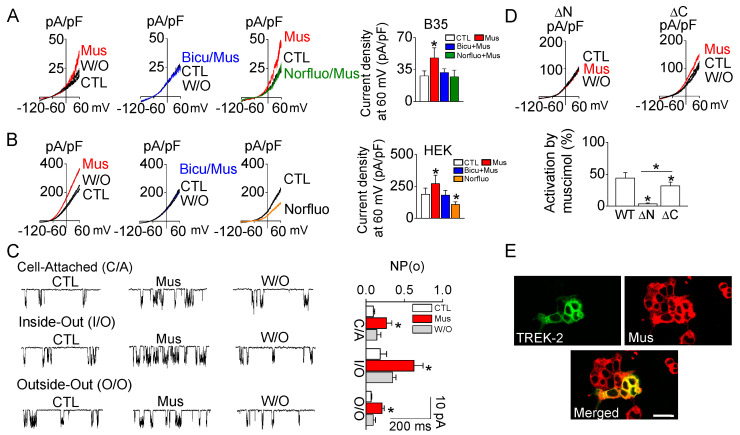
TREK-2 activation by muscimol. (**A**) Background K^+^ currents recorded in B35 cells. The currents were recorded in the presence of 4-aminopyridine (4-AP, 1 mM), BaCl_2_ (1 mM), and TEA (1 mM) to rule out the involvement of other K^+^ channels. (**B**) Muscimol-induced activation of TREK-2 currents in HEK-293 cells. TREK-2 was transfected into HEK293 cells. (**C**) Effect of muscimol on TREK-2 activation under different patch configurations. The muscimol was applied to the bath solution at a rate of 6 mL/min. The number of events analyzed for comparison of NPo was 2000. Channel activity was analyzed within 1 min after the application of muscimol at -60 mV. (**D**) The action sites of muscimol on the TREK-2 wild-type and N-terminal and C-terminal deletion (ΔN and ΔC) mutants. The whole-cell current was recorded in response to a voltage ramp (−120 to +60 mV; 1 s duration) from a holding potential of −80 mV. The currents measured at +60 mV were analyzed. (**E**) Binding assay of TREK-2 and muscimol. TREK-2 in the pcDNA3-EGFP vector was transfected into HEK-293 cells, and the BODIPY^®^ TMR-X muscimol conjugate was stained. Scale bar, 30 μm. Each bar (**A**–**D**) represents the mean ± SD of three independent experiments (*n* = 12). * *p* < 0.05 compared to the control.

In B35 cells, muscimol significantly increased the background current, and the muscimol-activated current was restored by washout and blocked in the presence of bicuculline (*p* < 0.05, Figure 4A). Norfluoxetine, a TREK-2 inhibitor [14], blocked muscimol-induced increase in the background current in B35 cells (Figure 4A). The currents were recorded in the presence of 4-aminopyridine (4-AP, 1 mM), BaCl_2_ (1 mM), and TEA (1 mM) to rule out the involvement of other K^+^ channels. The effect of muscimol on TREK-2 activity was evaluated in HEK-293 cells transfected with rat TREK-2. Muscimol (100 μM) application significantly increased TREK-2 currents by 1.5 ± 0.6-fold, whereas the muscimol effect was blocked in the presence of bicuculline (*n* =12, *p* < 0.05, Figure 4B). The inhibitory effect of norfluoxetine (30 μM) on TREK-2 currents was confirmed in the HEK-293 cells transfected with rat TREK-2 (Figure 4B). At +60 mV, muscimol at 10 μM, 30 μM, and 50 μM increased TREK-2 whole-cell currents by 1.9 ± 1.0%, 19.0 ± 5.1%, and 29.0 ± 3.9%, respectively, compared to control (*n* = 12). Muscimol activated the TREK-2 channels under the cell-attached, inside-out, and outside-out patches. The increase rate of the TREK-2 current by muscimol was the highest in the inside-out patch mode (C/A, 3.1 ± 0.3-fold; I/O, 3.5 ± 1.1-fold; O/O, 3.1 ± 0.9-fold; Figure 4C, each patch configuration; *n* = 12). There were significant differences between the three different patch modes (*p* < 0.05). The active site of muscimol was examined in the N-terminal and C-terminal deletion (ΔN and ΔC) mutants of TREK-2. The ΔN and ΔC mutants presented blockades and reduced muscimol effects compared to wild-type TREK-2, respectively (Figure 4D). The binding of muscimol to TREK-2 was assessed by treating TREK-2/GFP-transfected cells (green) with a BODIPY TMR-X muscimol conjugate (red). Red fluorescence was seen in all of the cells in the field, but no green fluorescence in all cells, indicating that some cells were not transfected with TREK-2. TREK-2 was expressed in the cytoplasm and cell membrane except for the nucleus. The green and red signals were colocalized (yellow signal), demonstrating that muscimol can bind to the TREK-2 channel (Figure 4E).

## 3. Discussion

To the best of our knowledge, this study is the first to report the likely correlation between GABA_A_R activation and TREK-2 channel activation.

### 3.1. Up-Regulation of TREK-2 Expression in GABAergic Neurons by Muscimol

GABAergic neurons suppress cellular excitability by converting the excitatory neurotransmitter glutamate to GABA, which causes Cl^-^ influx or K^+^ efflux, resulting in hyperpolarized membrane potential and reduced action potential. Many molecules present in GABAergic neurons are involved in regulating cellular excitability, and dysfunction of such molecules can affect GABA neurotransmission and eventually lead to various problems in the brain. Among these many molecules, ion channels are the most critical in cellular excitability. Alterations in the K_2P_ channel expression level are associated with neuronal function [15]. In the present study, the TREK/TRAAK channels were expressed in the GABAergic neurons in the mouse cortex, hippocampus, and medulla. GABAR agonists markedly upregulated the TREK-2 expression levels in B35 cells containing GABA and GABARs. GABAR activation increases the expression of various genes [16,17]. The TREK-2 gene (KCNK10) is one such gene. Dysfunction of these genes can lead to anxiety disorders [18], which may result from changes in neuronal maturation and development, synaptic production, and neurotransmitter release. TREK-2 upregulation has the potential to contribute to suppressing cell excitability and inducing hyperpolarization of membrane potential following GABAR activation.

TREK-1, which shares much genetic information and many electrophysiological and pharmacological properties with TREK-2, was largely localized to the nucleus of GABAergic neurons, unlike TREK-2 and TRAAK channels. The nuclear localization may not be clearly distinguishable in medullary GABAergic neurons derived from GAD67 mice, because nuclear signals were not tagged with nuclear dyes (see Figure 2). Several ion channels, including large conductance calcium-activated potassium (BK) channels, are expressed in the nucleus [19,20]. Nuclear ion chanels have a role in gene regulation via modulating nuclear Ca^2+^ signaling. They also play a role in the regulation of nuclear membrane potential and interact with other transcription-related proteins [19,20]. However, no report has shown that TREK-1 is only expressed in the nucleus. In screening with the cNLS Mapper, the rat TREK-1 protein has a putative nuclear localization sequence (NLS) with a score higher than 6.0. The rat TREK-2 protein has a NLS of less than 6.0, whereas the rat TRAAK protein has a NLS of zero. Higher scores indicate more active NLS. NLS is thought to be the cause of the nuclear localization signal in TREK-1. Further studies are needed to better understand the nuclear localization of TREK-1 and the difference in the expression patterns of TREK-1 and TREK-2. Ion channels with low expression in the nucleus may have a bigger function in the plasma membrane.

### 3.2. Activation of TREK-2 by GABA_A_ Receptor Agonists

Increased ion channel expression in cells does not imply increased channel activity. Even if ion channels are expressed, they will not be able to affect cell excitability or membrane potential unless they perform a function such as opening and closing. It has been reported that GABA_B_R activation inhibits neuronal excitability in the entorhinal cortex by activating TREK-2 channels [7]. In the present study, the GABA_A_R agonist muscimol enhanced TREK-2 expression more strongly than the GABA_B_R modulator, and the current was likewise more robustly triggered by muscimol. The increase in both the expression and activation of TREK-2 by muscimol means that the function of TREK-2 can be further enhanced. However, we have not fully elucidated the mechanism linking GABA_A_R activation to TREK-2 activation and its roles in the present study. The relationship between GABA_A_R activation and K^+^ channels has previously been reported in glial and cerebellar granule cells [21,22]. A previous study reported that muscimol (100 μM) inhibited the channel activity of the delayed rectifying and A-type K^+^ channels in cerebellar granule cells without a specific mechanism explanation [21].

Background currents activated by muscimol in B35 cells were blocked in the presence of bicuculline and the TREK-2 inhibitor norfluoxetine [14]. These results strongly indicate that the background K^+^ channel activated by muscimol is TREK-2. Muscimol highly activated currents at negative potentials in B35 cells compared to HEK-293 cells overexpressed with TREK-2, suggesting that B35 cells have other muscimol-responsive ion channels, such as Cl^-^ channels. Muscimol likely activates the TREK-2 channel along with other channels through GABA_A_R activation in B35 cells. TREK-2 channel activity was increased by muscimol in HEK-293 cells transfected with TREK-2, and the activity was blocked in the presence of bicuculline, indicating that the TREK-2 channel is related to the activation of GABA_A_R. However, there is no report indicating that HEK-293 cells express GABA_A_R. If HEK-293 cells do not express GABA_A_R, then muscimol seems to act directly on the TREK-2 channel. In particular, muscimol’s effect on TREK-2 channel activity was observed to be higher in the inside-out patch mode than in the cell-attached and outside-out patch modes. Moreover, muscimol did not affect the N-terminal deletion TREK-2 mutant (see Figure 4). Various experimental agents have been shown to either permeate the cell membrane or trigger specific intracellular events by binding to receptors when applied extracellularly [23]. Muscimol may act as other agents do. We propose that the site where muscimol can act is the N-terminus located on the cytoplasmic side of the TREK-2 channel. How can muscimol bind to the N-terminus in an intact cell? It would be possible if muscimol could permeate the plasma membrane. Muscimol may be able to increase TREK-2 currents by binding to the N-terminus of TREK-2 and activating a series of intracellular signals. Considering the indirect effect, muscimol binds to multiple binding sites of GABA_A_R extracellularly [24,25]. Specific intracellular signals may be activated to stimulate the N-terminus of TREK-2 after muscimol binds to the receptor in GABAergic neurons. TREK-2 activation is proposed as a likely mechanism by which muscimol decreases the excitability of GABAergic neurons. The specific mechanism, however, needs to be further investigated in brain slices.

### 3.3. The Physiological Role of TREK-2 Activation in GABAergic Neurons

Many medicines targeting GABA_A_Rs are commonly used as anesthesia and to treat anxiety disorders and insomnia [26]. GABA_A_R agonists generally depress brain activity by down-regulating neuroplasticity-related genes, activating the principal neuron population by suppressing local inhibitory regulation, and activating dopaminergic neurons via disinhibition [26]. GABA_A_R is a target for general anesthetics. Inhaled or volatile anesthetics hyperpolarize neurons via GABA_A_R activation [27] and K^+^ leak current activation [28]. TREK-2 could be also a target for such anesthetics. TREK channels are activated by the clinical concentrations of anesthetics such as chloroform, halothane, and isoflurane, and participate in the CNS actions of general anesthetics [29]. Several GABA_A_R agonists activate the TREK-2 channel. Wogonin, a benzodiazepine receptor ligand with an anxiolytic effect [30], transiently activates TREK-2 channels in both cell-attached and excised patches [31]. Agonists that activate GABA_A_R are likely to activate the TREK-2 channels in GABAergic neurons, and substances that activate TREK-2 are likely to play the role of GABAR agonists. GABA_A_R agonists may enhance their inhibitory functions through activating the TREK-2 channel in GABAergic neurons.

## 4. Materials and Methods

### 4.1. Chemicals

Unless otherwise stated, all chemicals were purchased from Sigma-Aldrich (St Louis, MO, USA). The GABA_A_ receptor agonist muscimol (100 mM), GABA_B_ receptor agonist baclofen (10 mM), and GABA_A_ receptor antagonist bicuculine (100 mM) were dissolved in 0.05 M HCl, 0.01 M HCl, and DMSO, respectively, to produce the stock solution. Norfluoxetine (10 mM) was dissolved in distilled water. All chemicals were diluted in the culture medium to their working concentration.

### 4.2. Cell Culture

The cell culture was performed as previously described [32]. B35 cells were purchased from American Type Culture Collection (ATCC, Manassas, VA, USA). Human embryonic kidney cell line HEK-293 cells were obtained from the Korean Cell Line Bank (Seoul, Korea). B35 cells and HEK-293 cells were cultured in Dulbecco’s modified Eagle’s medium (DMEM; Gibco/Life technologies, Grand Island, NY, USA) supplemented with 10% fetal bovine serum (FBS; Gibco), 100 U/mL penicillin (Gibco), and 100 mg/mL streptomycin (Gibco). The cells were incubated at 37 °C in 95% air and 5% CO_2_ gas mixture, and the medium was replaced once every two days.

### 4.3. Glutamate Decarboxylase 67 (GAD67)-GFP Knock-In Mice

Glutamate decarboxylase 67 (GAD67) is encoded by the GAD1 gene, which plays an important role in gamma aminobutyric acid (GABA) neurotransmission in the brain. GAD67-GFP knock-in mice (CB6-Tg (Gad1-EGFP) G42Zjh/J, #007677) were purchased from Jackson Lab (Bar Harbor, ME, USA). The characteristics of the Tg mice are described in detail on the Jackson Lab website (https://www.jax.org/strain/007677.html) [33,34]. A transgenic mouse line expressing enhanced green fluorescent protein (EGFP) in GABAergic neurons was constructed using the upstream regulatory sequence of the glutamate decarboxylase (GAD)1 gene (encoding the GABA synthesizing enzyme GAD67). Male mice (seven-week-old) were bred at the Gyeongsang National University Animal Center (GNUAC). Animal experiments were performed according to the Gyeongsang National Institute of Health guidelines and ARRIVE guidelines on the use of experimental animals. The mice were maintained under a 12 h light/dark cycle in a specific pathogen-free area with food and water freely available in the animal facility.

### 4.4. RT-PCR and Real-Time PCR

PCR was performed as previously described [32]. Total RNA isolated from B35 cells was used to synthesize the first-strand cDNA using a Superscript^TM^ II Reverse Transcriptase kit (Invitrogen, Carlsbad, CA, USA). First-strand cDNA, specific primers for the K_2P_ channels and GABA receptors, and Taq polymerase (Invitrogen) were used for PCR amplification. The sequences of the PCR primer pairs are listed in Table 1. Glyceraldehydes-3-phosphate dehydrogenase (GAPDH) was used as a loading control. The PCR conditions included initial denaturation at 94 °C for 5 min, followed by 30 cycles at 94 °C for 45 s, 55 °C for 30 s, and 72 °C for 2 min, with a final extension step at 72 °C for 5 min. The PCR products were electrophoresed on 1.5% (*w/v*) agarose gel to verify the product size. Images of the DNA fragments were directly captured using a gel imaging system with a digital camera (Canon, Tokyo, Japan) and ultraviolet transilluminator (Vilber Lourmat, Marne La Vallee, France). The expected bands were then extracted and directly sequenced with an ABI PRISM^®^ 3100-Avant Genetic Analyzer (Applied Biosystems, Foster City, CA, USA).

Changes in mRNA expression of K_2P_ channels (TASK-1, TASK-3, TREK-1, TREK-2, and TRAAK) were quantified by real-time PCR with a Fast-Start DNA Master SYBR Green I kit (Roche Diagnostics GmbH, Mannheim, Germany) and LightCycler^®^ capillaries (Roche). The PCR conditions for real-time PCR were as follows: initial denaturation at 95 °C for 10 min, 45 cycles for denaturation at 95 °C for 10 s, annealing at 55 °C for 10 s, and extension at 72 °C for 6 s. Primer efficiency was calculated with the slope value of the standard curve obtained from cycle threshold (Ct) and logarithmic (log) values of serial dilutions (stock, 1:10, 1:100, 1:1000, and 1:10,000 dilutions) of B35 cDNA. The primer set of the K_2P_ channel used in our experiment similarly showed about 92% efficiency. The 2^−ΔΔC^^t^ method was then used to calculate the relative levels of the K_2P_ channels’ mRNA [35], and the mRNA expression level was normalized to a reference gene (*GAPDH)*.

### 4.5. Immunostaining

Immunostaining was performed as previously described [32]. B35 cells were grown on round cover glass coated with poly-L-lysine for 24 h at 2.5 × 10^4^ cells/well (500 μL) in 24-well plates. After washing three times with 1× PBS, the cells were fixed with 4% paraformaldehyde in 0.1 M PBS for 30 min. The cells were then washed three times with 1× PBS for 10 min and pre-incubated in a blocking buffer containing 1% normal goat serum and 0.1% Triton X-100 for 2 h at room temperature. Brain tissues isolated from GAD67-EGFP Tg mice were fixed by perfusion with 4% paraformaldehyde, transferred to a 30% sucrose solution, and cut into 40 μm sections on a freezing microtome. The frozen tissue sections were fixed with 4% paraformaldehyde for 10 min at room temperature. After washing three times in PBS, the sections were incubated in a blocking buffer containing 1.5% normal goat serum in PBS for 1 h at room temperature. After blocking, the B35 cells and tissue sections were immunostained with anti-TREK-1, anti-TREK-2, and anti-TRAAK antibodies (1:200 dilution, Alomone Labs, Jerusalem, Israel) at 4 °C overnight. The cells and sections were then incubated with FITC-conjugated anti-rabbit immunoglobin G (IgG) and CY3-conjugated anti-rabbit IgG fluorescent secondary antibodies diluted to 1:400 in PBS for 2 h 30 min at room temperature. Propidium iodide (PI) was used for nucleus staining. The analysis was performed using a confocal laser scanning microscope (Olympus, Tokyo, Japan), and the negative control was analyzed by omitting the primary antibody.

### 4.6. Western Blot Analysis

Western blot analysis was performed as previously described [32]. B35 cells (2 × 10^5^ cells/60 mm dish) were treated with chemicals for 24 h. Total protein was isolated from the cells using RIPA lysis and an extraction buffer (Thermo Fisher Scientific, Waltham, MA, USA) containing 25 mM Tris-HCl (pH 7.4), 150 mM NaCl, 1% NP-40, 1% deoxycholate, 0.1% sodium dodecyl sulfate (SDS), and 1× protease inhibitor cocktail (Roche Diagnostics GmbH). The cell lysates were incubated for 30 min on ice with intermittent vortexing and then were clarified by centrifugation at 16,609× *g* (13,000 rpm, Hanil, Incheon, Korea) at 4 °C for 20 min. After centrifugation, the supernatant was separated and stored at −70 °C until use. Protein concentration in the cell lysates was quantified using a Pierce bicinchoninic acid (BCA) protein assay kit (Thermo Fisher Scientific). Equal amounts of proteins mixed with a 1× loading buffer among groups were separated on 12% SDS-polyacrylamide gel, and the gel was then blotted onto a polyvinylidene difluoride (PVDF, Millipore, Billerica, MA, USA) membrane for 15 min using semi-dry transfer (Bio-Rad, Hercules, CA, USA). Membranes were blocked with 5% (*w*/*v*) fat-free dry milk in tris-buffered saline with Tween-20 (TBST; 20 mM Tris HCl (pH 8.0), 137 mM NaCl, and 0.2% Tween-20) at room temperature for 60 min and then incubated with polyclonal anti-TREK-2 (1:500 dilution, Alomone Labs) and monoclonal anti-β-actin antibodies (1:5000 dilution) at 4 °C overnight. The primary antibody incubation was followed by incubation with secondary horseradish peroxidase (HRP)-conjugated goat anti-rabbit or anti-mouse antibody at 1:3000 (Assay Designs, Ann Arbor, MI, USA). Immuno-positive bands were visualized by enhanced chemiluminescence (Amersham ECL^TM^ Western Blotting Detections Reagent, Cytiva, Marlborough, MA, USA) according to the manufacturer’s instructions. Then, the membranes were exposed to the film and read using Developer and Fixer reagent solutions. The relative protein level was calculated using β-actin as a loading control.

### 4.7. Constructs

Rat TREK-2 cDNA (NM_023096) cloned in the pcDNA3.1 were kindly provided by Prof. Donghee Kim (Rosalind Franklin University of Medicine and Science, North Chicago, IL, USA). The wild-type TREK-2 was cloned in the pcDNA3-EGFP vector for image analysis. N-terminal deletion (ΔN) and C-terminal deletion (ΔC) constructs were generated by PCR against wild-type TREK-2, and the truncated constructs were cloned into pcDNA3.1 by a gene cloning service (COSMOgenetech, Seoul, Korea). The ΔN and ΔC constructs have deletions of 69 residues at the N-terminus and 217 residues at the C-terminus, respectively, based on previously reported TREK-2 sequence and membrane topology [36]. TREK-2 has a short N-terminus and a long C-terminus on the intracellular side [36].

### 4.8. Electrophysiological Studies

Electrophysiological recording was performed in B35 cells and TREK-2-transfected HEK-293 cells using a patch-clamp amplifier (Axopatch 200, Axon Instruments, Union City, CA, USA) according to a previous protocol [32]. HEK-293 cells were cultured on round cover glass coated with poly-L-lysin in a 35-mm dish for 24 h before transfection at a density of 2 × 10^5^ cells per 35 mm dish in DMEM containing 10% FBS (Invitrogen). HEK293 cells were transfected with rat TREK-2 cDNA cloned in pcDNA3.1 and GFP in pcDNA3.1 using LipofectAMINE (Invitrogen) and Opti-MEM^®^ I Reduced Serum Medium (Invitrogen). The green fluorescence from cells expressing GFP was detected using a Zeiss microscope (Jena, Germany) equipped with a mercury lamp as the light source. The cells were used 2–3 days after transfection. An electrophysiological study was performed as described previously [32]. Single-channel currents were filtered at 2 kHz using an 8-pole Bessel filter (−3 dB; Frequency Devices, Haverhill, MA, USA) and transferred to a computer (Samsung, Suwon, Korea) using a Digidata 1320 interface (Axon Instruments) at a sampling rate of 20 kHz. Threshold detection of the channel openings was set at 50%. In experiments using cell-attached and excised patches, the pipette and bath solutions contained (in mM) 150 KCl, 1 MgCl_2_, 5 EGTA, and 10 HEPES (pH 7.3). For whole-cell currents, the bath solution contained (in mM) 135 NaCl, 5 KCl, 1 CaCl_2_, 1 MgCl_2_, 5 glucose, and 10 HEPES, and the pipette solution contained (in mM) 150 KCl, 1 MgCl_2_, 5 EGTA, and 10 HEPES (pH 7.3). All solutions were prepared with Milli-Q water (18.2 MΩ-cm at 25 °C). The whole-cell current was recorded in response to a voltage ramp (−120 to +60 mV; 1 s duration) from a holding potential of −80 mV, and the currents measured at +60 mV were further analyzed using the pCLAMP program (version 10, Axon Instruments). Channel activity (NP_o_, where N is the number of channels in the patch, and P_o_ is the probability of a channel being open) was determined from data within 1 min of the current recording. All experiments were performed at ~25 °C.

### 4.9. TREK-2 Channel Binding to a Fluorescent Muscimol

HEK-293 cells transfected with TREK-2 in the EGFP vector were incubated for 20 min at room temperature in a physiological solution with 10 μM BODIPY^TM^ TMR-X-conjugated muscimol (Invitrogen). The cells were subsequently washed three times in a physiological solution, and the images were analyzed using a confocal laser scanning microscope (Olympus). The previously reported method was slightly modified and applied to our experiment [37].

### 4.10. Data Analysis and Statistics

Data analysis and statistics were performed as previously described [32]. The bands obtained from the RT-PCR and Western blot tests were quantified using the Sigma Gel image analysis software (version 1.0, Jandel Scientific, San Rafael, CA, USA) and Quantity One software (version 4.6.3, Bio-Rad) attached to a GS-800 calibrated densitometer (Bio-Rad). A Fluoview microscope (FV1000, Ver. 1.5, Olympus) was used to analyze immunofluorescence data. Data are represented as the mean ± SD. Significant differences between groups were analyzed using a one-way analysis of variance (ANOVA) with post-hoc comparisons using a Bonferroni test (OriginPro2020, OriginLab Corp. Northampton, MA, USA). *p* < 0.05 was considered as the criterion for statistical significance.

## 5. Conclusions

The GABA_A_R agonist muscimol increased TREK-2 expression levels in B35 cells and activated TREK-2 channels through indirect and direct mechanisms. The direct mechanisms emerged through the TREK-2 N-terminus. Our results suggest that muscimol-induced TREK-2 activation may contribute to cellular hyperpolarization and decreased cellular excitability in GABAergic neurons.

## Figures and Tables

**Table 1:** Gene primer sequences used for PCR.

Gene Name	GenBank Acc. No.	Primer Sequences (5′–3′)	Expected Size (bp)	Application
***GAPDH***	AF106860	F: CTAAAGGGCATCCTGGGC R: TTACTCCTTGGAGGCCATG F: CATGGCCTTCCGTGTTC R: CTGCTTCACCACCTTCTT	201 103	RT-PCR Real-time PCR
***Gabra1*** **(GABAA Receptor)**	L08490	F: ATCTTTGGGCCTGGACCCTC R: CGGGCTGGCTCCCTTGTCCA	580	RT-PCR
***Gabbr1*** **(GABAB Receptor)**	NM_031028	F: GTCTGGAGGAGGTGGTCGTT R: ACAAACGGGAACTGGCTTCT	534	RT-PCR
***Kcnk3*** **(TASK-1)**	NM_033376	F: TGTTCTGCATGTTCTACGCG R: TGGAGTACTGCAGCTTCTCG F: GAGCTGCCTAAGCGGTA R: AAAGTGCAGCATTTCAGCATA	702 142	RT-PCR Real-time PCR
***Kcnk9*** **(TASK-3)**	AF192366	F: TGACTACTATAGGGTTCGGCG R: AAGTAGGTGTTCCTCAGCACG F: GAAGTTTCTATGGAGAACATGGTGA R: CGTGGAAGAAGCTCCAAT	517 105	RT-PCR Real-time PCR
***Kcnk2*** **(TREK-1)**	AF325671	F: TGCCAAAGTGGAGGACACAT R: CTCTCCCACCTCTTCCTTCG F: TCTGAATGAATCAGAATGCTTTGCTA R: TCTGAATGAATCAGAATGCTTTGCTA	361 106	RT-PCR Real-time PCR
***Kcnk10*** **(TREK-2)**	AF196965	F: CAGCCCAAGAGTGCCACTAA R: GGATCCCAAAGATGGCGTAT F: TCAGTATGATTGGAGACTGGC R: ACTCAGCAGTGACATTAGC	493 107	RT-PCR Real-time PCR
***Kcnk4*** **(TRAAK)**	AF302842	F: CACCACTGTAGGCTTTGGCGATTATG R: ACTCTGCGTGTCTGAGGACTCGTCG F: CTGCCTGCTCTTTGTCC R: TACAGTGGTGAGTGTCACTATAAC	445 103	RT-PCR Real-time PCR
